# The paradox of cancer genes in non-malignant conditions: implications for precision medicine

**DOI:** 10.1186/s13073-020-0714-y

**Published:** 2020-02-17

**Authors:** Jacob J. Adashek, Shumei Kato, Scott M. Lippman, Razelle Kurzrock

**Affiliations:** 1 Department of Internal Medicine, University of South Florida, H Lee Moffitt Cancer Center and Research Institute, Tampa, FL 33612 USA; 2 Center for Personalized Cancer Therapy and Division of Hematology and Oncology, Department of Medicine, University of California San Diego Moores Cancer Center, Health Sciences Drive, La Jolla, CA 92093 USA

## Abstract

Next-generation sequencing has enabled patient selection for targeted drugs, some of which have shown remarkable efficacy in cancers that have the cognate molecular signatures. Intriguingly, rapidly emerging data indicate that altered genes representing oncogenic drivers can also be found in sporadic non-malignant conditions, some of which have negligible and/or low potential for transformation to cancer. For instance, activating *KRAS* mutations are discerned in endometriosis and in brain arteriovenous malformations, inactivating *TP53* tumor suppressor mutations in rheumatoid arthritis synovium, and *AKT*, *MAPK*, and *AMPK* pathway gene alterations in the brains of Alzheimer’s disease patients. Furthermore, these types of alterations may also characterize hereditary conditions that result in diverse disabilities and that are associated with a range of lifetime susceptibility to the development of cancer, varying from near universal to no elevated risk. Very recently, the repurposing of targeted cancer drugs for non-malignant conditions that are associated with these genomic alterations has yielded therapeutic successes. For instance, the phenotypic manifestations of CLOVES syndrome, which is characterized by tissue overgrowth and complex vascular anomalies that result from the activation of *PIK3CA* mutations, can be ameliorated by the PIK3CA inhibitor alpelisib, which was developed and approved for breast cancer. In this review, we discuss the profound implications of finding molecular alterations in non-malignant conditions that are indistinguishable from those driving cancers, with respect to our understanding of the genomic basis of medicine, the potential confounding effects in early cancer detection that relies on sensitive blood tests for oncogenic mutations, and the possibility of reverse repurposing drugs that are used in oncology in order to ameliorate non-malignant illnesses and/or to prevent the emergence of cancer.

## Background

In recent years, the rate of development of small molecule and antibody drugs that effectively target oncogenic drivers has increased rapidly [1, 2]. The natural question that emerges is whether or not targeting these genomic alterations in non-malignant illness could also have salutary effects, as there are (i) benign conditions (including but not limited to seborrheic keratosis, endometriosis, arteriovenous malformations in the brain, and Alzheimer’s disease) that arise sporadically and that harbor somatic mutations that are believed to be drivers in cancer (Table 1), and (ii) germline and hereditary phenotypes and somatic mosaic phenotypes that are associated with such mutations (e.g., achondroplasia, neurofibromatosis, CLOVES syndrome, and Proteus syndrome) (Table 2). The benign disorders that harbor putative “oncogenic drivers” have a variable propensity for malignant transformation and, in the case of hereditary conditions that are caused by such mutations, patients have differing vulnerabilities for the development of malignancy, ranging from minimal or no increased risk to a very high lifetime susceptibility to cancer.

**Table 1:** Examples of sporadic benign conditions, many with negligible potential for malignant transformation, associated with somatic alterations in driver cancer genes

Gene	Type of alteration	Benign or premalignant condition	Frequency of alteration in benign condition (%)	Examples of drug(s) that can potentially target the alteration	Examples of malignancies associated with this gene alteration	Mechanism
*BRAF*	V600E, D594V, V599E	Melanocytic nevi	70–88% [3–12]	BRAF and/or MEK inhibitors such as dabrafenib and trametanib [13, 14]	Melanoma	RAS-RAF-MEK-ERK pathway upregulation [15]
*NRAS*	Q61K	Giant congenital melanocytic nevi	6–14% [10, 11]	MEK inhibitors [12] such as trametinib [16]	Melanoma	RAS-RAF-MEK-ERK pathway upregulation [15]
	Q61K and Q61R	Melanocytic nevi	70–95% [17, 18]	MEK inhibitors such as trametinib [16]	Melanoma	RAS-RAF-MEK-ERK pathway upregulation [15]
*FGFR3*	R248C, S249C, G372C, S373C, A393E, K652E, K652M	Seborrheic keratosis	∼ 18–85% [19–22]	FGFR inhibitors such as erdafitinib [23]	Urothelial carcinoma	Activation of the FGF/FGFR machinery [24]
	R248C, G372C, G382R	Epidermal nevi	33% [25]	FGFR inhibitors such as erdafitinib [23]	Urothelial carcinoma	Activation of the FGF/FGFR machinery [24]
*PIK3CA*	E542K, E545K, H1047R	Seborrheic keratosis	∼ 16% [20]	PIK3CA inhibitors such as alpelisib [26]	Breast cancer	PI3K-AKT-mTOR pathway activation
	M1043V	Endometriosis	~ 4% [27]	PIK3CA inhibitors such as alpelisib [26]	Breast cancer	PI3K-AKT-mTOR pathway activation
	H1047L, H1047R	Normal esophagus mucosa	Not listed [28]	PIK3CA inhibitors such as alpelisib [26]	Breast cancer	PI3K-AKT-mTOR pathway activation
*ALK*	TPM3-ALK, TPM4-ALK	Inflammatory myofibroblastic tumor	∼ 50% [29]	ALK inhibitors [30] such as alectinib [31]	Non-small cell lung cancer	ALK pathway activation [32]
*NOTCH1*	Loci not specified	Aging esophagus	12–80% [33]	No specific inhibitors approved	Colon cancer	Wnt-beta-catenin pathway activation [34]
*KRAS*	G12V or G12D	Arteriovenous malformations in brain	∼ 63% [35, 36]	MEK inhibitors such as trametinib [16]	Colorectal and pancreatic cancer	RAS-RAF-MEK-ERK pathway upregulation [15]
	G12C, G12V, G12A, G12D, G12R	Endometriosis	~ 21% [27]	MEK inhibitors such as trametinib [16]	Colorectal and pancreatic cancer	RAS-RAF-MEK-ERK pathway upregulation [15]
	Q61R	Normal testis	Not listed [28]	MEK inhibitors such as trametinib [16]	Colorectal and pancreatic cancer	RAS-RAF-MEK-ERK pathway upregulation [15]
*TP53*	R177S, Q192L, R196*, K139R, H193Y, E224fs, N239S	Rheumatoid arthritis synovium	17–46% [37, 38]	Bevacizumab may target angiogenesis upregulation that results from *TP53* mutations [39]	Serous ovarian cancer (*TP53* mutations are common across cancers)	*TP53* is a tumor suppressor gene [40]
	Loci not specified	Aging esophagus	2–37% [33]	Bevacizumab may target angiogenesis upregulation that results from *TP53* mutations [39]	Serous ovarian cancer (*TP53* mutations are common across cancers)	*TP53* is a tumor suppressor gene [40]
*CTNNB1*	T41A and S45P	Desmoid tumor	88% [41]	COX-2 inhibitors [42] such as celecoxib [43], as well as sorafenib (which can suppress CTNNB1-mediated activation of the WNT pathway) [13, 14, 44]	Adrenocortical cancers	Wnt-beta-catenin pathway activation [45]
*FGFR2*	Y376C, P286S	Keratinocytic epidermal nevus	5–10% [46]	FGFR inhibitors such as erdafitinib [23]	Urothelial carcinoma	FGF/FGFR machinery [24]
*AKT*, *MAPK*, and *AMPK* pathway genes	–	Alzheimer’s disease	~ 27% [47]	mTOR inhibitors or MEK inhibitors	Multiple tumor types	Increases tau phosphorylation

**Table 2:** Examples of hereditary germline syndromes and of somatic mosaicism associated with examples of alterations in cancer-driver genes, their relationship with cancer in affected patients, and targeted drugs that might be useful

Gene	Alteration	Syndrome	Descriptions	Increased incidence of cancer (if yes, most common cancers)	Treatment potentially/theoretically targeting the alteration
*APC*	Most common nonsense changes are C>T mutations [48]	Familial adenomatous polyposis [49]	Multiple non-cancerous (benign) growths (polyps) in the colon with strong predisposition to cancer	Yes (colorectal [49, 50])	Sorafenib and WNT inhibitors [13, 44]
*ARAF*	S214P [51]	Central conducting lymphatic anomaly [52]	Not listed	None found	mTOR inhibitors such as sirolimus [53] or MEK inhibitors such as trametinib [51]
*BRAF*	Q257R, S467A, G596V, V600G	Cardiofaciocutaneous syndrome [54]	Cardiac abnormalities, distinctive craniofacial appearance, and cutaneous abnormalities	Yes (juvenile myelomonocytic leukemia, brain tumors, acute lymphoblastic leukemia, rhabdomyosarcoma, and neuroblastoma [55])	BRAF inhibitors [9] and/or MEK inhibitors such as dabrafenib [5] and cobimetinib [7]
	G469E, F595L, L597V	Noonan syndrome [56, 57]	Unusual facial features, short stature, heart defects, bleeding problems, and skeletal malformations	Yes (juvenile myelomonocytic leukemia, brain tumor, acute lymphoblastic leukemia, rhabdomyosarcoma, and neuroblastoma [55])	–
*ERBB4*	R927Q, R1275W	Amyotrophic lateral sclerosis subtype 19 [58]	Degeneration of motor neurons and anterior horns of spinal cord	None found	Pan-ERBB inhibitors such as neratinib [59] will not be effective because the mutations have an inactivating effect
*FGFR1*	L165S, L191S	Hartsfield syndrome [60]	Holoprosencephaly, ectrodactyly, and cleft lip/palate	None found	These FGFR1 mutations may cause loss of function, so FGFR inhibitors such as erdafitinib [23] will not be effective
	Multiple loss of function mutations	Kallman syndrome [61]	Hypogonadotropic hypogonadism and impaired sense of smell	None found	–
	P252R	Pfeiffer syndrome [62]	Premature fusion of certain skull bones	None found	Gain-of-function alterations and hence may be targeted by FGFR inhibitors such as erdafitinib [23]
*FGFR2*	S252W or P253R	Apert syndrome [63]	Premature fusion of certain skull bones (craniosynostosis*) and syndactyly	Hepatoblastoma [64]*	Mutations are gain of function and hence may be targeted by FGFR inhibitors such as erdafitinib [23]
	Y375C or S372C	Beare-Stevenson cutis gyrata syndrome [65]	Premature fusion of certain skull bones (craniosynostosis*)	Hepatoblastoma [64]*	–
	S351C	Pfeiffer syndrome [62]	Premature fusion of certain skull bones (craniosynostosis*)	Hepatoblastoma [64]*	–
*FGFR3*	G380R; R248C, G372C, G382R	Achondroplasia [66]	Short-limbed dwarfism	None found	Mutations are gain of function and hence may be targeted by FGFR inhibitors such as erdafitinib [23]
	N540K	Hypochondroplasia [67]	Short-limbed dwarfism that is milder than achondroplasia	None found	–
	D513N	Lacrimo-auriculo-dento-digital syndrome [68]	Abnormal tear production, malformed ears with hearing loss, decreased saliva production, small teeth, and hand deformities	None found	–
	P250R	Muenke syndrome [69]	Craniosynostosis*, hearing loss, subtle hand and foot abnormalities, and developmental delay	Hepatoblastoma [64]*	–
	R248C, K650E, S249C, Y373C	Thanatophoric dysplasia [70]	Extremely short limbs and folds of extra (redundant) skin on the arms and legs	None found	FGFR3 inhibitor in mice [71]
*GNAS*	R201C, R201H, Q227L	McCune-Albright syndrome [72]	Abnormal scar-like (fibrous) tissue in their bones, a condition called polyostotic fibrous dysplasia	Yes (breast, thyroid, testicular [73])	MEK inhibitors [74] such as trametinib [75]
*HRAS*	G12S, G12C	Costello syndrome	Delayed development/intellectual disability, loose folds of skin, unusually flexible joints, and distinctive facial features including a large mouth, heart problems	Yes (juvenile myelomonocytic leukemia, brain tumor, acute lymphoblastic leukemia, rhabdomyosarcoma, and neuroblastoma [55])	MEK inhibitors [76] such as trametinib [75]
*IDH2*	R140Q	D-2-hydroxyglutaric aciduria [77]	Delayed development, seizures, weak muscle tone (hypotonia), and abnormalities in the cerebrum	Yes (high-grade glioma [78])	IDH2 inhibitors such as enasidenib [79]
*JAK3*	R651W, V599G, W709R	Severe combined immunodeficiency [80]	Lack the necessary immune cells to fight bacteria, viruses, and fungi	None found	Mutations cause loss of function and hence JAK inhibitors such as tofacitinib [81] will not be effective
*KRAS*	P34R	Cardiofaciocutaneous syndrome [54, 82]	Distinctive craniofacial appearance, and cutaneous abnormalities (including but not limited to xerosis, hyperkeratosis, pigmented moles, hemangiomas)	Yes (juvenile myelomonocytic leukemia, brain tumor, acute lymphoblastic leukemia, rhabdomyosarcoma, and neuroblastoma [55])	MEK inhibitors [83] such as trametinib [75]
*MET*	F841V	DFNB97 hearing loss [84]	Non-syndromic sensorineural hearing loss with prelingual onset	None found	The mutation is damaging, so MET inhibitors such as cabozantinib [85] should not be effective
*NOTCH1*	C1496Y, D1989N	Adams-Oliver syndrome [86]	Congenital aplasia cutis and malformations of the limbs	None found	Loss-of-function mutations so Notch inhibitors such as LY3039478 [87] will be ineffective
*NF1*	R304X, Y2264X, R1825W, R1809C, N1229S, D176E	Neurofibromatosis type 1 [88]	Changes in skin coloring (pigmentation) and the growth of benign neoplasms along nerves in the skin, brain, and other parts of the body [89]	Yes (malignant peripheral nerve sheath tumors, optic gliomas, brain tumors, breast cancer [90])	MEK inhibitors [91] such as trametinib [75] or selumetinib [92]
*NF2*	L46R, L141P, A211D, K413E, Q324L, and L535P	Neurofibromatosis type 2 [93]	Growth of benign neoplasms in the nervous system; vestibular schwannomas or acoustic neuromas	None found	mTOR inhibitors [94] such as sirolimus [53]
*RET*	P155L, T278A, T278P, D300N, S316I, C620R	Hirschsprung disease [95]	Absence of nerves in distal colon	Yes (medullary thyroid [96, 97])	Mutations generally cause loss of function, so RET inhibitors such as LOXO-292 [98] or cabozantinib [83] would be ineffective; RET C620R may cause both gain and loss of functions
*STK11*	40 different somatic *STK11* mutations [99]	Peutz-Jegher syndrome	Gastrointestinal hamartomatous polyps and hyperpigmentation of the lips, buccal mucosa, digits	Yes (gastrointestinal tract, pancreas, cervix, ovary, and breast [100])	mTOR inhibitors such as everolimus [101]
*TP53*	Multiple loss of function mutations	Li-Fraumeni [102–105]	Greatly increases the risk of several cancers	Yes (sarcoma, breast, brain, adrenocortical [102])	Bevacizumab may target angiogenesis associated with *TP53* mutations [39]
Somatic mosaicism					
*AKT1*	E17K (gain of function)	Proteus syndrome [106]	Overgrowth of the bones, skin, and other tissues	Yes (meningiomas, ovarian cystadenomas, breast cancer, parotid monomorphic adenoma, mesothelioma [107])	AKT inhibitors such as ipatasertib [108]
*GNAQ*	R183Q	Sturge-Weber syndrome [109]	Port-wine stains affecting the skin, leptomeningeal vascular malformations	None found	Some MEK inhibitors may have activity
*PIK3CA*	E545K	Hemimegalencephaly [110]	Rare neurological condition in which one-half of the brain, or one side of the brain, is abnormally larger than the other	None found	PIK3CA inhibitors such as alpelisib [24]
	H1047R, C420R, Q542K	CLOVES syndrome [111]	Tissue overgrowth and complex vascular anomalies; CLOVES stands for congenital lipomatous (fatty) overgrowth, vascular malformations, epidermal nevi and scoliosis/skeletal/spinal anomalies	Yes (Wilms tumor [112])	PIK3CA inhibitors such as alpelisib [26, 113]
	H1047R and H1047L	Fibroadipose hyperplasia [114]	Patchy overgrowth of a limb or part/region of the body	None found	PIK3CA or mTOR inhibitors [115] such as alpelisib [26] or everolimus [101]

*A recent publication [64] shows that craniosynososis may be associated with increased incidence of hepatoblastoma, although the authors did not define which syndromes were affected

**Fig. 1:**
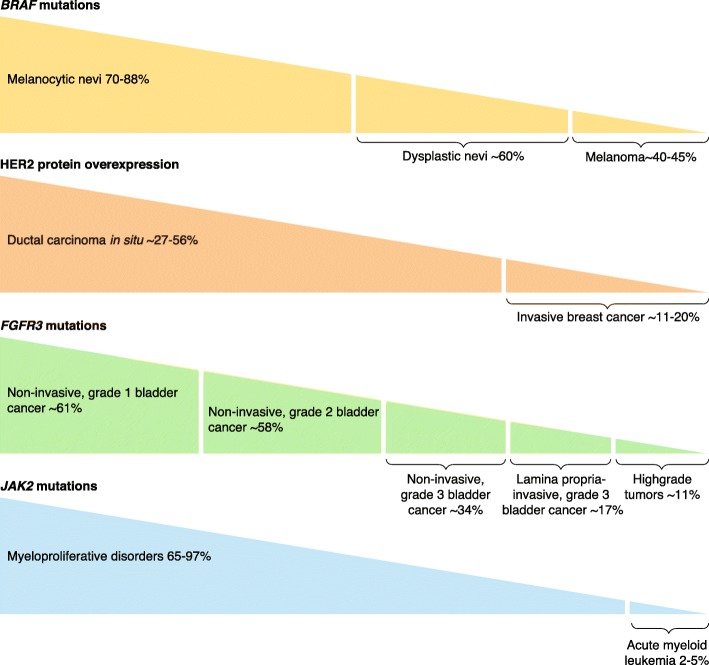
Examples of reverse clonal selection. Aberrant cancer drivers that are paradoxically more frequent in benign or premalignant counterparts than they are in the malignant condition. *BRAF* mutations included V600E [3, 4, 8, 117–120] and HER2 overexpression [121–123]. *FGFR*3 mutations included R248C, S249C, and G372C [124–126]. *JAK2* mutations included *V617F* [127–129]. *% given* is the percentage of cases in which there are alterations (e.g., 70–88% of melanocytic nevi have *BRAF* mutations)

Interestingly, there is also growing evidence that the canonical theory of renegade clonal expansion in carcinogenesis [116] may not be the only manner in which malignant development proceeds. The theory of clonal expansion posits that clones of cells harboring oncogenic drivers will be selected during the development of malignancy because these driver(s) confer a growth advantage. Hence, the percentage of cells with the oncogenic driver(s) will be smaller in premalignant lesions than in lesions that are malignant. However, the opposite is sometimes found (Fig. 1). For instance, *BRAF* V600E driver mutations are discerned at twice the frequency in benign nevi, which do not transform to melanoma, than in melanoma itself [3, 4, 117]. This paradoxical phenomenon has also been reported in the continuum from benign to malignant in other diseases (Fig. 1).

There are several important consequences of “oncogenic drivers” in benign conditions. First, there are the implications for early detection of cancer based on sensitive blood tests that assess circulating cell-free DNA (cfDNA) [130–132]. If mutations identical to those found in cancer also occur in conditions with no malignant predisposition, their presence may confound the early diagnosis of cancer premise that is the basis of these blood-based screening tests, such as the multi-cancer detection blood test developed by GRAIL that has been granted breakthrough status by the US Food and Drug Administration [133].

Furthermore, as mutations that are indistinguishable from those in cancer exist in benign conditions, and as drugs are available that effectively neutralize the impact of these mutations in cancer, it is plausible that these drugs could be repurposed for illnesses other than cancer. Indeed, several such examples have been established in preclinical models and in patients. For instance, it has recently been demonstrated that increased expression of PARP1, a well-known anti-apoptotic cancer target, plays a role in neuronal cell death in Parkinson’s disease. Consequently, it has been suggested that PARP inhibitors, which have successfully been used to treat *BRCA*-mutated cancers [134–137], merit examination as candidate drugs in Parkinson’s disease [138]. In *BRCA-*mutated tumors, repair of double-stranded DNA breaks is deficient. PARP1 is a protein that is important for repairing single-strand breaks; and the suppression of PARP catalytic activity by PARP inhibitors further compromises DNA repair, resulting in tumor cell lethality. In Parkinson’s disease, PARP is elevated and causes alpha-synuclein spread, triggering cell death and Parkinson’s symptoms; theoretically, PARP inhibitors could reverse this process.

Another example in which a drug developed for cancer can be used in a non-cancer condition is provided by CLOVES syndrome, which is caused by mutations in *PIK3CA*. Patients with CLOVES syndrome, which manifests as congenital lipomatous overgrowth, vascular malformations, epidermal nevi, and scoliosis/skeletal and spinal anomalies, also have a propensity to Wilms tumors [112]. CLOVES syndrome can be treated with the PIK3CA inhibitor alpelisib, which was developed for *PIK3CA*-mutant breast cancer [113].

In this review, we provide an overview of and update on the rapidly expanding knowledge regarding the conundrum of oncogenic drivers in benign disorders, and we discuss the profound implications of these findings for the treatment of both benign and malignant conditions [139]. First, the ubiquitous finding of oncogenic drivers in non-malignant conditions may prove problematic for the development of sensitive blood tests for early detection of cancer. Second, non-malignant conditions that are caused by actionable oncogenic drivers could potentially be treated with repurposed drugs that have been successfully developed to target and manage cancers harboring those drivers. Examples of such effective repurposing already exist, suggesting that the molecular alterations found in benign disease are indeed drivers of benign disease (as they are in cancer) and not “uninvolved bystanders.” Such strategies are especially important because some of the benign conditions in which these mutations have been found are rare or ultra-rare and present a huge unmet therapeutic need. Importantly, some non-malignant conditions are associated with an increased risk of malignant transformation because of their underlying oncogenic driver. In such conditions, an approach aimed at deploying anti-cancer drugs to target molecular alterations in benign disease might also be exploitable to prevent cancers.

## Sporadic benign conditions associated with alterations in “driver” cancer genes

With the advances in next-generation sequencing (NGS) and the resulting identification of driver mutations for various cancers, there has been growing interest in the phenomenon in which well-known cancer-causing genes are altered in benign conditions, some of which have either no (or very limited) potential for malignant transformation (Table 1): (i) *FGFR3* activating mutations are well documented to play a major role in the pathogenesis of bladder cancer [124–126], yet they are also found in unrelated conditions such as seborrheic keratosis and epidermal nevi [19–22]; (ii) mutations in the *TP53* tumor suppressor gene, which are perhaps the most common alterations in cancer [140], also characterize the synovium of rheumatoid arthritis [37, 38]; (iii) *KRAS* mutations are found in arteriovenous malformations [35, 36, 141] and in endometriosis [27] (though their functional role is still unclear in these conditions); and (iv) brain somatic mutations in Alzheimer’s disease, in which about 27% of patients (14 of 52) have alterations in genes of the PI3K-AKT, MAPK, and AMPK pathways, are known to contribute to hyper-phosphorylation of tau [47]. Importantly, some of the loci that are mutated in each of these cases do not differ from the loci that are mutated and implicated in cancer. The mechanism by which such mutations cause these benign conditions but fail to cause cancer is unclear, but we hypothesize that aberrant tissue growth that is associated with *FGFR3* mutations is dependent on the tissue- or cell-type context of these mutations; when they are found in the epidermis, benign seborrheic keratosis develops [19–22], whereas when they appear in the bladder, cancer develops [124–126]. A similar mechanism could be posited for *KRAS* mutations and arteriovenous malformations. In the case of *TP53* mutations, which are clearly related to the formation of multiple cancers [140], perhaps they induce the inflammatory condition rheumatoid arthritis, rather than cancer, in the synovium [37, 38]. Arthritis might ensue because *TP53* mutations upregulate levels of the inflammatory cytokine interleukin-6 (IL-6), a known pathogenic factor in rheumatoid arthritis [142, 143].

An example that defies the tissue- or histology-context hypothesis is *BRAF* V600E, a known oncogenic driver that occurs in around 80% of benign nevi [3, 4]. These nevi are extremely common and are estimated to have a transformation-to-melanoma rate of less than 0.03% for melanocytic nevi [144] and only about 4.8% for dysplastic nevi [145]. Yet, in the setting of melanoma or other tumors, there can be no doubt regarding the oncogenic role of *BRAF V600E* mutations, based on preclinical modeling [146] and also on the tumor regression that results from the use of antagonists such as BRAF and MEK inhibitors [5, 75]. Explanations for the lack of pathogenicity of *BRAF V600E* in benign nevi include, but are not limited to the following: (i) RNA silencing, a mechanism whereby deleterious DNA alterations are not expressed at the RNA level [147]; or (ii) the possibility that a “double hit” [148], a concomitant loss of an inhibitor or the activity of a genomic co-factor [149], is necessary in order to initiate carcinogenesis. Another possible mechanism by which oncogenic mutants can exist in normal tissue but not cause cancer is illustrated by a study that showed that normal human esophagus contains *TP53-*mutant progenitors. Yet, *TP53*-mutant cells can be displaced from normal tissues through the improvement of the competitive fitness of wild-type progenitors by antioxidants [150].

Both normal aging and specific environmental exposures can also be associated with somatic oncogenic mutations. For instance, in natural aging of the esophagus and in rapidly proliferating tissues such as those in the testes, mutations in known oncogenes such as *NOTCH1* [33], *PIK3CA* [28], *TP53* [33], and *KRAS* [28] may appear. Indeed, in middle-aged and elderly persons, it was found that cell clones containing cancer-associated mutations covered much of the esophageal epithelium, with *NOTCH1* and *TP53* mutations affecting 12–80% and 2–37% of cells, respectively [33]. The progressive age-related expansion of clones that carry mutations in driver genes in the esophagus can be accelerated substantially by smoking and by alcohol consumption. Although the remodeling of the esophageal epithelium by driver-mutated clones is an inevitable part of normal aging, lifestyle risks may also affect cancer development [151]. Somatic mutations also emerge in skin that is exposed to ultraviolet light [152]. Indeed, aged, sun-exposed skin is a patchwork of thousands of evolving clones with over 25% of cells harboring cancer-causing mutations while preserving epidermal functions. Similarly, age-associated clonal hematopoiesis, which is caused by acquired mutations in myeloid cancer-associated genes such as *DNMT3A* or *TET2*, is highly prevalent in the normal population. Its biological impact on hematopoiesis, etiology, and oncogenic risk is poorly delineated at this time [153–156]. Finally, probable driver mutations have been reported in around 1% of normal colorectal crypts in middle-aged adults, indicating that carcinomas are rare outcomes despite a pervasive process of neoplastic change in morphologically normal colorectal tissue [157]. The degree to which the size of the mutant clones influences risk of malignant progression warrants further exploration [158].

A critical question as regards mutations that arise with aging, or as a result of exposure to smoking or other noxious environmental factors, relates to the mechanisms that promote or prevent cancer development. Immune surveillance may play an important role in explaining the presence of oncogenic drivers in benign conditions without progression to malignancy. It could be postulated that natural immune mechanisms may eradicate cells that present neo-antigens derived from these mutations. Failure of this immune surveillance might result in cancer. Indeed, findings in both mouse models of cancer and humans with cancer offer compelling evidence that immune cell types and effector pathways collectively function as potent tumor suppressor mechanisms [159, 160]. Furthermore, it has been shown that the ability of various individuals’ major histocompatibility complexes to present neo-antigens that are produced by the mutanome shapes the mutational landscape in cancers and may predict each patient’s susceptibility to specific tumors [161].

In summary, oncogenic drivers are found in a range of benign conditions as well as in normal tissues, especially with aging. Their limited transformation potential or failure to induce cancers consistently [157] can be hypothesized to be due to several reasons including, but not limited to, tissue and cellular context, a need for genomic driver co-factors or for co-loss of genomic suppressors, the suppressive or competitive growth of progenitors with normal molecular landscapes, the size of the mutant clones, and immune surveillance.

## Hereditary conditions that result from germline cancer-related genes have a range of malignant potential

Cancer-associated genes can be altered at the germline level, and yet individuals with these genes may have a wide spectrum of cancer risk, from no increased risk to very high risk (Table 2). It is unclear as to why there is a range of cancer susceptibility, but this range could be related to immune surveillance mechanisms [161]. As an example, patients with “RASopathies” (a group of rare genetic conditions such as cardiofaciocutaneous syndrome and Costello syndrome caused by mutations in genes of the RAS-RAF-MAPK pathway) have an increased risk of juvenile myelomonocytic leukemia, brain tumors, acute lymphoblastic leukemia, rhabdomyosarcoma, and neuroblastoma [55]. These patients do not, however, have increased risk of classic *BRAF*-mutated melanoma, although ~ 75% of the cardiofaciocutaneous syndromes result from germline *BRAF* mutations [162], and pigmented nevi are very distinct in this syndrome and help to define it [163].

In other familial syndromes, such as Von Hippel-Lindau, patients harbor a *VHL* mutation, which has been best defined in clear cell renal cell carcinoma, and subsequently are at significant risk of developing renal cancers [164]. Li-Fraumeni syndrome is another example of a hereditary cancer syndrome in which *TP53* mutations predispose patients to cancers of the breast, brain, or adrenocortical organ, or to sarcomas [102]. Further, the *APC* gene mutation is a well-defined and known cause of familial adenomatous polyposis, and afflicted individuals are at significant risk of developing colorectal carcinoma [49, 50].

On the other hand, there are hereditary conditions caused by “oncogenic driver mutations” that have no clear association with increased cancer risk (although large-scale studies of these diseases are not fully developed and it is conceivable that, with time, some increased cancer risk might be identified). Examples include achondroplasia, hypochondroplasia, lacrimo-auriculo-dento-digital syndrome, and thanatophoric dysplasiam, each of which is attributed to germline *FGFR3* mutations that result in their varied phenotypes (Table 2). Patients with neurofibromatosis type 2 also seem to have no clear association with an increased cancer risk [93].

In summary, germline oncogenic mutations are associated with a variety of aberrant phenotypes and a wide spectrum of increased cancer risk (ranging from negligible to very high). The reasons for the variance in vulnerability to malignancies are unclear but could involve the immune machinery [159–161, 165]. It is also possible that heterozygosity may, in some cases, play an antagonistic role in tumor initiation and malignant transformation (even while accelerating the formation of benign neoplasms), as shown for *NF1* [166]*.* Patients who carry some of these germline oncogenic alterations need to be monitored, often throughout their lifespan, for specific cancers on the basis of their diagnosis and the known propensity to malignancy, with cancer risk being determined by epidemiologic studies.

## Somatic mosaic conditions that are associated with oncogenic drivers but without clear increased cancer risk

Somatic mosaicism is defined by the occurrence of two genetically distinct populations of cells within an individual, derived from a postzygotic mutation [167]. Unlike inherited mutations, somatic mosaic mutations may affect only a portion or a tissue of the body and are not transmitted to offspring. The phenotypic consequences of somatic mosaicism are dependent upon the biologic impact of the mutation, as well as on the developmental time at which the mutation occurs and the areas of the body that are affected [168].

Several somatic mosaic conditions are associated with gene abnormalities identical to those in cancer but result in a phenotypic presentation other than cancer (Tables 2 and 3). Sturge-Weber syndrome is a neurocutaneous vascular malformation syndrome, characterized by a facial port-wine birthmark, which is associated with choroid “angioma” of the eye and malformed leptomeningeal blood vessels, as well as with seizures, strokes, stroke-like episodes, and neurologic deficits, beginning in infancy [109]. It is caused by a somatic (not heritable) mosaic mutation in *GNAQ*. This activating mutation in *GNAQ* (R183Q) results in constitutive overactivation of the Ras-Raf-MEK-ERK pathway and is identical to the GNAQ alteration implicated in uveal melanoma [173, 174]. It has been hypothesized that the occurrence of the GNAQ mutation at a different time in development (in the fetal period or in infancy rather than in adulthood) accounts for its resulting in a vascular malformation rather than a cancer [175].

**Table 3:** Examples of sporadic and hereditary conditions and of somatic mosaic non-malignant conditions that have been treated successfully in animal models or in patients by targeting underlying “oncogenic” drivers using drugs, some of which were developed for cancer

Condition	Underlying molecular defect	Therapy	Result of therapy	Comments	FDA-approved drug: cancers treated
Sporadic conditions					
Rheumatoid arthritis	*TP53* mutations	Tocilizumab, which is an anti-IL-6 receptor antibody	Decreased incidence of flares, better disease control [169]	Efficacy in humans; *TP53* mutations are known to increase IL-6, which mediates inflammation [142]	None
Desmoid tumors	*CTNNB1* mutations	COX-2 inhibitors and sorafenib	Tumor regression [5, 145, 146]	Efficacy in humans; COX-2 inhibitors and sorafenib can abrogate the activation of the WNT pathway by *CTNNB1* alterations [13, 41, 42]	COX-2 inhibitors: none Sorafenib: renal cell carcinoma, hepatocellular carcinoma
Inflammatory myofibroblastic tumors	*ALK* rearrangements	Crizotinib	Sustained objective responses [30]	Efficacy in humans; crizotinib is a potent ALK inhibitor	Non-small cell lung cancer
Schnitzler syndrome	*MYD88* L265P mutation	Anakinra, which is an IL-1 antagonist	Complete remission of disease [170]	Efficacy of anankinra in humans	None
Neurofibromatosis 1	*NF1* mutations	MEK inhibitor selumetinib	71% partial response rate for inoperable plexiform neurofibromas [92]	FDA granted breakthrough status for selumetinib for NF1 in 2019	None
Hereditary and somatic mosaic conditions					
CLOVES syndrome	Mosaic gain-of-function *PIK3CA* alterations	Alpelisib, which is PIK3CA inhibitor	Improved disease-related symptoms [113]	Efficacy in humans	Hormone-positive, HER2-negative breast cancer
Central conducting lymphatic anomaly	Gain-of-function *ARAF* mutations (MEK or mTOR pathway)	Sirolimus (mTOR inhibitor) or trametinib (MEK inhibitor)	Resolution of chylous output over the course of a week with removal of chest tube with sirolimus (*n* = 1) [53] Dramatic clinical improvement, with remodeling of the patient’s lymphatic system and resolution of the lymphatic edema, marked improvement in pulmonary function tests, cessation of supplemental oxygen requirements and near normalization of daily activities with trametinib (*n* = 1) [51]	Efficacy in humans	Sirolimus: none Trametinib: melanoma
Fibroadipose hyperplasia	*PIK3CA* mutations	Sirolimus (mTOR inhibitor)	Stabilization or improvement in disease in patients [115, 171]	Efficacy in humans	None
Achondroplasia	*FGFR3* mutations	FGFR3 inhibitor in mouse models	Restored size of embryonic achrondroplastic femurs in animals [172]	Animal model efficacy	None

Fibroadipose hyperplasia is characterized by patchy overgrowth of a limb or of a part or region of the body. It is associated with *PIK3CA* H1047R mutations, which are implicated in multiple cancers [114, 115, 171]; yet, this condition is not known to associate with cancer, although further longitudinal studies are necessary. Hemimegalencephaly, a condition in which one side of the brain is larger than the other, is also attributed to an activating *PIK3CA* E545K that is indistinguishable from the alteration observed in several types of malignant neoplasms, but there is no clear cancer risk in hemimegalencephaly [176, 177].

In summary, as for conditions that are associated with germline mutations, conditions caused by somatic mosaic mutations may be associated with aberrant tissue growth and with a range of cancer risks (Table 2). Cancer risk may relate to the actual mutation involved, tissues affected and developmental period, and to other poorly studied factors such as immune function. Because these conditions are very rare, it is conceivable that more in-depth investigations of them will reveal some increased cancer risks, even in those conditions that are currently not believed to carry such a risk. Epidemiological surveys are needed in order to define cancer risk in these disorders fully. However, such studies may be challenging because of the rarity of the disorders. Finally, for patients who have elevated cancer risk, lifetime monitoring for the specific cancers that are most likely to occur is needed.

## The paradox of reverse clonal evolution and selection

The classic theory of clonal evolution and selection posits that driver alterations cause cancer progression from benign to premalignant lesions and then to invasive malignancy (Fig. 1). Indeed, cancers are believed to evolve by a reiterative process of clonal expansion, genetic diversification, and clonal selection within the adaptive backgrounds of tissue bionetworks [178]. Clonal evolution involves the interplay of advantageous or “driver” alterations that give a cancer cell a fundamental growth advantage, genomic alterations that enhance the rate of other DNA changes by creating genomic instability (“mutator” genes), neutral or “passenger” (hitchhiker) gene alterations that do not directly determine cancer development, and modifications to the tumor habitat that refashion the fitness effects of each of these abnormalities [179–181]. The dynamics are complex, with highly variable configurations of genetic diversity and ensuing clonal architecture. Further, evolutionary selection pressures that operate at a multicellular level—and therefore can be distinct from the clonal events that drive initiation and the benign-to-malignant transition—govern late-stage tumor progression and metastases [116, 182]. These issues are important because therapeutic interventions are aimed at driver alterations, which must be distinguished from passenger mutations. It has been previously assumed that hotspots, meaning sites in the genome that are prone to mutations across multiple tumors, are drivers of tumorigenesis; however, it has been demonstrated more recently that many hotspot mutations represent passenger events, recurring at sites that are simply more predisposed to mutation [183]. Impacting driver mutations may decimate cancer clones and their ecosystems, but may also provide potent selective pressure for the emergence and/or expansion of resistant molecular alterations [116].

A canonical understanding of clonal evolution and selection suggests that driver alterations should appear more frequently as the continuum progresses from benign to premalignant to malignant neoplasm. Traditionally, it would be assumed that, for example, a *BRAF V600E* mutation—identified as a known driver of melanoma on the basis that mutated BRAF proteins have elevated kinase activity and are transforming in NIH3T3 cells [117]—would be found most abundantly in melanomas rather than in dysplastic or benign nevi. On the contrary, however, the incidence of the *BRAF V600E* mutation in benign nevi and premalignant conditions or dysplastic nevi is more frequent (~ 70–88% and ~ 60%, respectively) than in melanoma (~ 40–45%) (Fig. 1), despite the fact that the conversion rate of benign nevi to melanoma is negligible [144]. Another example that contradicts the classic theory of clonal expansion is HER2 overexpression, a clearly druggable driver of breast malignancies, which is nonetheless identified more commonly in ductal carcinoma in situ (~ 27–56%) than in invasive mammary cancers (~ 11–20%) [121–123]. Similarly, grade of bladder cancer is inversely related to the frequency of driver *FGFR3* mutations. As successive grades are diagnosed, the incidence of *FGFR3* mutations decreases: non-invasive, grade 1 bladder cancer has the most frequent occurrence of *FGFR3* mutations (~ 61%), then non-invasive, grade 2 bladder cancer (~ 58%), followed by non-invasive, grade 3 bladder cancer (~ 34%), lamina propria-invasive grade 3 (~ 17%), and, last, high-grade tumors, which demonstrate *FGFR3* mutations in only about 11% of cases [124–126]. This paradoxical phenomenon is also seen in hematologic malignancies. *JAK2* mutations are found in the majority of myeloproliferative disorders (65–97%), but rarely in acute myeloid leukemias (2–5%) [127–129, 184, 185]. In each of the examples mentioned above, there can be little question regarding the driver role of these alterations because of the efficacy of drugs developed against them in achieving tumor regression.

The mechanism that underlies the paradoxical decrease in the frequency of driver alterations with malignant progression is unknown. However, the phenomenon is especially pertinent to therapeutic drug development because it is critical that one does not assume that a mutation or other alteration is a passenger just because it is more frequently found in the benign counterpart of an invasive cancer. Had such an assumption been made, BRAF inhibitors would not have been developed for melanoma. Another question is how oncogenic drivers that are less frequent in malignant disease than in benign disease act to impart the oncogenic phenotype in the malignancy, but not in the benign lesions. Perhaps the driver alteration acts in an oncogenic capacity only when a required co-factor or co-alteration is in place, or perhaps the suppression of an endogenous inhibitor is required in order for the malignancy to emerge [186]. Preclinical and ex vivo studies examining the functional effects of mutations in various tissue contexts and with different co-alterations can be performed with a variety of techniques, including patient-derived cell cultures that serve as avatars [187]. These studies may provide a biologic understanding of the role of these mutations in determining the aggressiveness of a tumor, and whether or not malignant transformation takes place.

## Therapeutic implications of oncogenic drivers in non-malignant conditions

In many instances, there are approved drugs that specifically target a gene mutation product and are readily available for use in the setting of a malignancy. Using the same gene-targeting paradigm and shifting it towards sporadic benign diseases, hereditary conditions or somatic mosaic syndromes that carry the cognate driver genomic aberration (regardless of their malignant potential) could offer innovative treatments for these conditions, perhaps reversing their phenotype. Factors that would need to be considered would be the potency of the agent against the genomic target and its potential toxicity. For disorders that have potential for malignant transformation, it is conceivable that the use of such targeted agents might also attenuate the risk of developing cancer.

### Repurposing cancer drugs for sporadic conditions

Several examples now exist to demonstrate how the targeting of genomic drivers in benign illnesses can alleviate disease, and to show that drugs that were developed for illnesses on the neoplastic spectrum can be used (Table 3). For instance, tocilizumab is an anti-IL-6-receptor monoclonal antibody approved for use in rheumatoid arthritis and also developed for the treatment of Castleman disease, a lymphoma-like condition [169]. *TP53* mutations, which are known to occur in the synovium in rheumatoid arthritis [37, 38], upregulate IL-6 levels [142, 143], perhaps mediating the inflammation of arthritis and explaining the efficacy of tocilizumab in this condition. Desmoid tumors provide another example; these neoplasms are an aggressive fibromatosis that have similarities to fibrosarcoma but are considered benign because they do not metastasize. They are characterized by *CTNNB1* mutations [41], which are known to activate the WNT pathway [13]. They can be treated with COX-2 inhibitors such as celecoxib (approved for familial adenomatosis polyposis, which predisposes carriers to colorectal cancer) and/or with sorafenib (approved for several types of cancer), both of which suppress the WNT pathway [14, 42, 43].

Another example is inflammatory myofibroblastic tumor, which is an uncommon, usually benign neoplasm composed of myofibroblastic spindle cells with an inflammatory infiltrate. Approximately half of inflammatory myofibroblastic tumors carry rearrangements of the anaplastic lymphoma kinase gene locus (*ALK*) on chromosome 2p23, causing aberrant ALK expression. After the initial report of a striking response to treatment with the ALK inhibitor crizotinib (approved for lung cancers with *ALK* rearrangements) in a patient suffering from an *ALK-*rearranged inflammatory myofibroblastic tumor [30], a larger study showed that six of 12 ALK-positive patients (50%) achieved an objective response with crizotinib [188].

Finally, in Schnitzler syndrome, a rare auto-inflammatory disease that often presents with urticarial rash, fever, lymphadenopathy, musculoskeletal pain, and thrombosis and that is attributed to cytokine dysregulation involving IL-1β and the inflammasome pathway, there is evidence that blocking IL-1 can lead to significant disease control [170]. We previously described a patient with Schnitzler syndrome and a *MYD88* mutation; the latter is classically discerned in Waldenström macroglobulinemia. Treatment with anakinra, an IL-1 receptor antagonist (IL-1RA), resulted in a durable response [170]. This beneficial effect may be due to the fact that *MYD88* plays an important role in IL-1 signaling, mediating the association between IL-1R- and the IL-1R-associated kinase (IRAK) [189].

Theoretical examples also exist. For instance, drugs that target PIK3CA or MEK signals, such as alpelisib or trametinib, respectively, may theoretically offer new options for women suffering with endometriosis, which harbors mutations in *PIK3CA* or *KRAS* [27]. In sporadic brain arteriovenous malformations (AVMs) that are caused by *KRAS* mutations, using agents that inhibit the MAP-ERK pathway could also offer potential therapy for patients, at least in theory [35]. These AVMs have potential to rupture and cause significant morbidity in these patients.

Taken together, these observations suggest that drugs that impact driver molecular alterations or their downstream effectors can be repurposed to treat a variety of benign, sporadic illnesses, and that such new uses merit investigation in clinical trials that select drugs for non-malignant conditions on the basis of their somatic alterations. Nevertheless, several caveats would need to be considered. These include the possibility that the drug action might depend on tissue context and that potential side effects might attenuate the ability to administer the drug to patients who are afflicted with non-malignant conditions.

### Repurposing cancer drugs for somatic mosaic and germline conditions

Gene-product targeted drugs may also be beneficial in hereditary or somatic mosaic conditions (Table 3). A dramatic example is provided by CLOVES syndrome (congenital lipomatous overgrowth, vascular malformations, epidermal nevi, scoliosis/skeletal, and spinal syndrome), which is a disorder that results from somatic, mosaic gain-of-function mutations of the *PIK3CA* gene and that belongs to the spectrum of *PIK3CA*-related overgrowth syndromes. Previously, this ultra-rare condition had no specific treatment and a poor survival rate. Use of the PIK3CA inhibitor alpelisib improved disease-related symptoms in all of the 19 patients that received the drug [113]. Intractable vascular tumors became smaller, congestive heart failure was improved, hemihypertrophy was reduced, and scoliosis was attenuated. The treatment was not associated with significant toxicity at doses of alpelisib of 250 mg by mouth per day in adults taken for a period of up to 18 months (the approved dose for breast cancer starts at 300 mg per day); children received 50 mg per day with excellent tolerance.

A second illustration of the repurposing of medications has been described in patients with central conducting lymphatic anomaly, in which aberrations can occur along the MAPK or mTOR pathways [52]. The use of sirolimus (a mTOR inhibitor) [53] or trametinib (a MEK inhibitor) [51] provided significant benefit and attenuation of disease in treated patients. For example, a patient given sirolimus, who required a chest tube for the abundant output of chylous effusion, attained a complete resolution of chylous output and no longer required the chest tube [53]. In the patient treated with trametinib, there was resolution of the lymphatic edema, improvement on pulmonary function tests so that the patient no longer required supplemental oxygen, and significant improvement in functional status [51]. In other words, the phenotype of these genetic disorders was reversed by precise targeting of the molecular abnormality using a drug developed for cancer.

Another example pertinent to the repurposing of drugs for benign illness pertains to *NF1*, a gene whose aberration activates the MEK pathway. Neurofibromatosis-1 is a hereditary condition caused by germline *NF1* mutations; it manifests mainly with non-malignant neurofibromas, which nonetheless cause functional disabilities. Recently, the MEK inhibitor selumetinib was given Breakthrough Status by the FDA for this condition because of a ~ 70% response rate in children with neurofibromatosis-1 and inoperable plexiform neurofibromas [92]. Of interest, *NF1* mutations may also be found in melanoma, but some studies suggest that targeting them with MEK inhibitors would be ineffective (though there may be exceptions) [190]. Melanomas with NF1 mutations may not respond to MEK inhibitors (although neurofibromatosis is responsive) because melanomas tend to have important co-alterations, whereas neurofibromatosis is driven only by *NF1* alterations [191, 192].

Finally, targeting activating *FGFR3* mutations in achondroplasia with FGFR inhibitors is another example worth noting, although the data here are from animal models only [23]. In a mouse model with *FGFR3*-mutated skeletal cells, use of an FGFR3 inhibitor led to restoration in the size of achrondroplastic femurs [172]. *FGFR* mutations cause multiple skeletal disorders and also play a role in certain cancers. Targeting these mutations could potentially abrogate the skeletal anomalies seen in these hereditary conditions. However, if the lack of increased cancer risk in these patients is due to a compensatory factor that develops in the presence of germline activated *FGFR3*, and if this compensatory factor is attenuated in the presence of FGFR inhibitors given during early life stages, it would be important to take into consideration the theoretical possibility of a later cancer risk if these FGFR3 inhibitors were discontinued [193].

## Confounding the holy grail—early detection of cancer with blood tests

In recent years, liquid biopsy to detect cfDNA or circulating tumor DNA (ctDNA) has emerged as an attractive non-invasive methodology to discern cancer-specific genomic aberrations in plasma. Numerous studies have reported the utility of ctDNA in advanced cancer [194–197]. In particular, ctDNA assays can capture a more global portrait of tumor heterogeneity than that provided by tissue DNA (which reflects the small piece of tissue that is biopsied rather than DNA shed from both primary and multiple metastatic sites [198]); therefore, ctDNA can be exploited to monitor tumor response and resistance.

Recently, ctDNA analysis has also been proposed as a promising future tool for the identification of early neoplasms as part of cancer screening. As the average amount of mutated DNA in plasma is very low (about 0.4% even in metastatic malignancies), exceedingly sensitive technologies must be developed; further, in cancer patients with low tumor burden, ctDNA is difficult to detect [130, 199]. Hence, in patients without known tumors who are being screened, the levels of ctDNA may be very, very low. Yet, increased sensitivity of ctDNA tests is a two-edged sword. It is plausible that with overly sensitive tests, molecular alterations from benign lesions would be picked up in cfDNA. Being able to differentiate between these sources of ctDNA and to determine thresholds that correspond to levels of concern for screening tools are areas of continuing development [200]. It is also possible that serial tests may need to be conducted and that increasing ctDNA levels with time might be the trigger for further work up for cancer. In addition, as cancers are heterogeneous at the molecular level, any screening blood test would need to assay multiple gene targets in order to increase the chances of finding a cancer.

Of significant interest, non-invasive prenatal testing, which uses cfDNA as an analyte to detect copy-number alterations in the fetal genome (by testing maternal blood), can detect early cancers in pregnant women. In one study, an abnormal genomic profile not consistent with fetal abnormalities was identified in about 10 out of 100,000 cases; a significant subset of these observations (18 of 43; 41.9%) was attributed to mostly unsuspected maternal malignant neoplasms [201]. These findings substantiate the claim that sensitive cfDNA screening may be exploitable as a cancer biomarker for the early detection of malignant disease.

In addition to cfDNA or ctDNA, other components of tumors that are shed into the circulation may be important for early detection: circulating tumor cells or extracellular vesicles. Indeed, these tumor components have been informative for early recognition of relapse, albeit of advanced tumors [202].

For the identification of early cancer, strategies for analysis are in principle relatively similar to those for advanced disease. However, beyond the sensitivity issues discussed above (i.e., very early-stage (asymptomatic) tumors may not release enough ctDNA to be detectable in a typical blood draw), the challenges with these techniques are considerable. For instance, white blood cells are a major source of cfDNA in blood, and it is crucial to distinguish acquired mutations in leukocytes (benign clonal hematopoiesis that increases with age [203]) from incipient invasive cancer. Further, “oncogenic” mutations can be found in healthy individuals, including in their cfDNA, and can be indistinguishable from those associated with cancer [130]. Therefore, caution needs to be applied when interpreting results from mutation-based early detection tools, as both false negatives (resulting from lack of sensitivity) and false positives (resulting from the detection of shed DNA from benign lesions that harbor oncogenic mutations) could confound the interpretation of these tests. Other methods being explored to screen for cancers using blood-based methods include the use of autoantibodies [204–208] and tumor-associated antigens [209]. As regards technologies that use circulating tumor cells or extracellular vesicles, in addition to the low volume of the aberrations in the blood, theoretically confounding phenomena must be addressed. These might include the rate of clearance in patients with renal or hepatic impairments, stability in the bloodstream, diurnal or other biologic influences on time of collection, the effects of smoking, pregnancy, and other inflammatory conditions, and clonal expansions of non-tumors.

Other technologies, including gene and protein expression signatures [210–214], have also been developed to help to decipher the code that differentiates benign and cancerous molecular anomalies. Intriguingly, there are models that predict (with up to 90% accuracy) the pattern of epigenetic changes found on circulating DNA in the bloodstream that imply malignancy versus those that do not [215]. Indeed, there is evidence that the methyl clusters that occur on the cancer DNA not only help to identify cancer DNA, but are major contributors to carcinogenesis [215].

In summary, myriad blood-based assays are being developed for early detection of cancer. They include tests of ctDNA mutations or methylation patterns as well as interrogation of exosomes or circulating tumor cells. Validating these biomarkers will probably require serial follow-up to discern an increasing level of abnormality and will also need threshold trigger values for imaging patients in order to confirm the presence of cancer.

## Perspective and future directions

The rapid expansion of the use of NGS in cancer clinical care and research has resulted in significant improvement in outlook for a subset of malignancies [216–218]. Indeed, genomic markers can drive new clinical trials of both gene- and immune-targeted agents [219–225]. Relatively new, however, is the emergence of data showing that non-cancerous illnesses also have genomic markers, and intriguingly, that some of these molecular alterations are indistinguishable from those considered oncogenic drivers for certain malignancies. Further large-scale studies across benign conditions may provide insight into crucial, subtle differences in the molecular landscape that enable the same “driver” to navigate towards two different “destinations”—that is, benign versus malignant disease. Identifying potential co-alterations may be key; alternatively, it may be that tissue of origin or histologic context is critical or that immune function shapes the outcome.

A wide variety of sporadic, mosaic, and hereditary conditions can be characterized by “oncogenic” aberrations, including conditions that have negligible malignant potential (Tables 1, 2, and 3). Furthermore, there are now several examples of the paradox of decreasing frequency of the “oncogenic driver” as the condition progresses from benign to premalignant to malignant (Fig. 1). Importantly, recent RNA sequence analysis also identified the somatic clonal expansion of mutations associated with cancer across normal tissues, most commonly in the lung, skin, and esophagus; the number of mutations correlated with age and with tissue proliferation rate [28]. The presence of these molecular abnormalities in benign conditions may confound efforts to detect cancer event cascades early through the use of blood tests. Serial blood tests may need to be done, with increasing levels of the biomarker being indicative of a cancer concern.

Of significant future interest is the potential to repurpose drugs used in cancer for non-malignant illnesses that harbor actionable genomic alterations and/or to prevent the development of cancer in conditions and syndromes where there is a predisposition to malignancy. The use of open-label basket clinical trials, in which patients are matched with drugs on the basis of a genomic aberration (regardless of histology), has been effective in a variety of cancer settings [16, 226–229]; similar approaches could conceivably be taken in benign conditions, for which trials that are disease agnostic could be developed and drug choice would be dictated by the genomic aberration. Alternatively, individual sequencing studies of somatic or germline tissue may define the treatment prosecution strategy on an N-of-one basis in selected non-malignant diseases, as it is beginning to do in malignancy [223]. Regardless, patients would require close follow-up to determine whether their cancer risk was modified by the use of matched targeted agents, and functional studies on tissues might help to identify those conditions that are most likely to respond to cognate compounds. Finally, moving forward in this field will require multidisciplinary collaborative teams with expertise in the benign conditions, their malignant counterparts, and targeted drugs and genomics, as well as translational scientists to bridge the emerging preclinical and clinical data.

## Abbreviations

*ALK*: Anaplastic lymphoma kinase gene locus
AVM: Arteriovenous malformation
cfDNA: Circulating cell-free DNA
ctDNA: Circulating tumor DNA
FDA: US Food and Drug Administration
IL: Interleukin
NGS: Next-generation sequencing
